# Role of Self-Control and Cognitive Ability in Dietary Habits, Physical Activity, and Body Mass Index Among Medical Undergraduates

**DOI:** 10.7759/cureus.83271

**Published:** 2025-04-30

**Authors:** Uzma Zafar, Zaima Ali, Attiqa Khalid

**Affiliations:** 1 Physiology, Lahore Medical and Dental College, University of Health Sciences and University of Biological and Applied Sciences, Lahore, PAK

**Keywords:** cognitive ability, cognitive functioning self-assessment scale, dietary habits association, physical activity, self-control

## Abstract

Introduction

Self-control and cognition are mental processes that influence reasoning, modulate actions and shape decision-making. Both these processes regulate lifestyle choices, such as dietary habits and physical activity. Understanding the connection between self-control and cognition in conditioning dietary behavior and physical workouts is vital for developing targeted approaches to enhancing overall well-being. The present study explores the associations of self-control and cognition with dietary habits, physical activity, and body mass index (BMI).

Methods

This cross-sectional study was conducted at a private medical college, over a ten-month period. The study population consisted of MBBS students across all the academic years. The study tool was a questionnaire with five domains. Domain 1 included demographic characteristics, Domain 2 the ‘Brief Self-Control Scale,' Domain 3 the ‘Cognitive Functioning Self-Assessment Scale,' Domain 4 the ‘Fat and Fiber Behavior Questionnaire,’ and Domain 5 utilized the ‘Global Physical Activity Questionnaire.’

Results

The data collection form was distributed to 480 students, of whom 400 completed and submitted it, yielding a response rate of 83%. Data was entered and analyzed by SPSS version 26 (IBM Corp., Armonk, New York). Out of 400 students, 120 (30%) were males and 280 (70%) females. On chi-square test, there was significant association of self-control with body mass index (p=0.001), dietary habits (p=0.01), and cognition (p=0.001). Body mass index also showed a significant association with dietary habits (p=0.03). However, there was no significant association of cognitive level with body mass index (p=0.894), dietary habits (p=0.994), and physical activity (p=0.664). Binary logistic regression was conducted to assess the association between BMI and variables, including physical activity, self-control, dietary habits, and cognition, while controlling for age and gender. Higher self-control was significantly associated with lower odds of obesity (p=0.003). Dietary habits showed a potential protective effect, but the association was not statistically significant (p=0.07). Cognition and physical activity were not significantly associated with BMI (p>0.05).

Conclusion

These findings underscore the potential importance of self-control in weight management, suggesting that self-control may be more promising for supporting a healthier body mass index compared to cognitive function or physical activity. Longitudinal studies are needed to confirm the predictive value of self-control on body mass index over a span of time.

## Introduction

Self-control and cognition play fundamental roles in defining dietary behavior and physical activity, which exert a significant impact on body mass index (BMI). Self-control, the quality to delay gratification and manage impulses, is the key factor for sustaining balanced eating habits and committing to healthful physical activity [1]. Subjects with higher self-control traits are likely to resist temptations for unhealthy food, comply with structured diet plans, and adhere to regular physical activity routines. Alternatively, low self-control is associated with impulsive reckless eating, frequent indulgence in high-calorie meals, and a sedentary routine, resulting in higher BMI and a greater risk of obesity [2].

Cognition is a major psychological factor for the effective accomplishment of tasks. It incorporates processes such as decision-making, attention, and higher-order thinking, which also affect nutritional patterns and workout habits. Cognitive capabilities enable individuals to make wise dietary choices, plan healthy meals, and appreciate the long-term benefits of staying active [3]. Cognitive ability is also intimately related to health literacy, which can lead to effective care of personal and community well-being. There is an interconnected role of healthy dietary habits, physical activity, brain health, and cognitive functioning. The cognitive skill of dietary awareness guides in making healthy food choices, maintaining a balanced diet, and sustaining physical activity. People with higher cognition often have access to better job opportunities, healthcare and housing, which can contribute to overall performance and accomplishments. Conversely, low cognition may result in unhealthy dietary plans, susceptibility to calorie-dense food, and reduced commitment to physical exercise [4].

Cognition and self-control are mental functions incorporating actions and reasoning, modulating approaches, managing thought processes, and influencing decision-making. While cognition and self-control are closely connected, they play a distinct part in shaping daily behavior. Cognition encompasses the mental processes of learning, analyzing, decision-making, and problem-solving. Self-control primarily includes resisting temptations, managing emotions, and delaying gratification for long-term gains. Both cognition and self-control influence risk-taking behavior and lifestyle choices such as diet and physical activity [5,6].

Understanding the connection between self-control and cognition in conditioning dietary behavior and physical exercise is vital for developing targeted approaches aimed at enhancing overall well-being. By improving self-regulatory traits and cognitive abilities, individuals may be better equipped to adopt lasting health habits, ultimately mitigating the effects of chronic diseases [6]. The present study explores the associations of self-control and cognition with dietary habits, physical activity, and BMI.

## Materials and methods

This cross-sectional study was conducted at a private medical college in Lahore over a period of ten months from March to December 2024. Approval was taken from the Institutional Review Board (Ref. No. LMDC/19821/23). Informed consent was obtained from the students and they were briefed about the purpose of the study.

The study population consisted of MBBS students across all academic years. The sampling technique was convenience sampling. The sample size was calculated by the online open source and free software OpenEpi using the equation for cross-sectional descriptive study [7]. The level of significance was chosen as 0.05. The power of the study was set as 80%. The minimum sample size calculated was 372, with a confidence interval of 95%. The present study included 480 participants.

Prior to participation, written informed consent was obtained from the students and they were briefed about the purpose of the study. All the students who gave their consent were included in the study. All those not present on the day of the survey were excluded.

Before the distribution of the questionnaire, height was measured using a stadiometer, and participants were standing upright on a flat surface without shoes. Body weight was measured using a calibrated digital weighing scale. BMI was calculated by dividing weight in kilograms by height in meter square [8].

The study tool was a questionnaire with five domains. Domain 1 included demographic characteristics, Domain 2 the ‘Brief Self-Control Scale (BSCS),’ Domain 3 the ‘Cognitive Functioning Self-Assessment Scale (CFSS),’ Domain 4 the ‘Fat and Fiber Behavior Questionnaire (FFBQ),’ and Domain 5 utilized the ‘Global Physical Activity Questionnaire’ (GPAQ). The questionnaire was distributed to 480 students, of whom 400 completed and submitted it, yielding a response rate of 83%. Participation was voluntary, and respondents were requested to complete and return the questionnaire within 30 minutes (see Appendix, Table 5).

A hybrid approach of data collection was used that combined self-administration and researcher facilitation. It allowed participants to fill out the questionnaire independently while providing the option for guidance and clarification if needed. Self-control was assessed by the ‘Brief Self-Control Scale (BSCS)’ a psychometric tool designed and developed by Tangney et al. [9]. This scale focuses on the main aspects of self-control, including self-discipline, acting without thinking, and impulse control. The test-retest reliability of BSCS is 0.87 [9-11]. The BSCS consists of a concise set of 13 items assessed on a five-point Likert scale, ranging from not at like me to very much like me (total score range is 13-65). Nine of these items were reverse-scored. A higher total score indicates greater self-control.

Cognitive ability was measured by using the Cognitive Functioning Self-Assessment Scale (CFSS), a self-reported measure designed to assess various cognitive domains such as attention, memory, and spatial-temporal orientation. The scale comprises 18 items, each rated on a five-point Likert scale, ranging from 1 ("never") to 5 ("always"). The test-retest reliability and Cronbach’s alpha coefficient for the scale were reported as 0.79 and 0.86, respectively [12,13]. A higher score on the scale reflects greater impairment in cognitive functioning (total CFSS score range is 18-90).

The Fat and Fiber Behavior Questionnaire (FFBQ) is a 20-item instrument designed to assess dietary behaviors related to fat and fiber intake [14]. It comprises 13 items focusing on fat-related behaviors and seven items assessing fiber intake. The questionnaire demonstrates high test-retest reliability, with intra-class correlations (ICCs) ranging from 0.87 to 0.89. Responses generate three indices: fat index, fiber index, and total index. The total index is calculated by dividing the total score with a number of responses. The score range is 1 (least healthy) to 5 (most healthy). A cutoff value of 3 was established, with scores of 3 or above indicating a fiber-rich diet or healthy behavior, while a score below 3 reflects a fat-rich diet or unhealthy behavior [14-16].

Physical activity was assessed using the Global Physical Activity Questionnaire (GPAQ) by the World Health Organization, with modifications to the scoring criteria to align with the specific objectives of this study. The GPAQ was feasible to administer and can be scored as a continuous or categorical variable. The reliability coefficient of this questionnaire was 0.83 [17,18]. GPAQ collected information on three domains [19]. These were activities at work, travel to and from places at home, and recreational activities. The total score ranged from 6 to 30. In this study, a cutoff value of 16 was used. Subjects with a score equal to or above 16 were classified as active, while those with a score below 16 were categorized as less active.

Data from each response was entered and analyzed using SPSS Version 27.0 (IBM Corp, Armonk, New York). Frequencies and percentages were given for qualitative variables. Mean±standard deviation was given for quantitative variables. The chi-square test was applied to determine the association of study variables such as self-control, cognition, BMI, dietary behavior, and physical activity. A p-value of less than 0.05 was considered significant. Binary logistic regression analysis was conducted to identify significant independent variables influencing BMI. The dependent variable was BMI, while the independent variables included physical activity, self-control, dietary behavior, and cognition. Confounder variables, such as age and gender, were controlled in the model. A p-value of less than 0.05 was considered statistically significant.

## Results

The questionnaire was distributed to 480 students, of whom 400 completed and submitted it, yielding a response rate of 83%. The present study included 400 participants. The descriptive characteristics of the participants are given in Table 1.

**Table 1:** Descriptive characteristics of the study population.

Continuous variables	Mean	Standard deviation
Self-control score	42.67	7.35
Cognitive ability score	44.01	9.05
Dietary index score	2.76	0.53
Physical activity score	16.13	5.97
Body mass index	23.64	2.13
Age	20.99	1.71

On the basis of the mean score of BSCS, CFSS, FFBQ, and physical activity, operational definitions of study variables are given below.

A cutoff value of 43 was established for BSCS, with scores above 43 indicating optimal self-control, while scores of 43 or below denote suboptimal self-control. The BSCS is a 13-item self-report metric of self-control. Higher scores indicate a higher self-control level. The cut-off level 43 on the Brief Self-Control Scale was established based on the distribution of scores within our study population. The mean score in this study was 42.67±7.35. The cut-off level 43 in this study is a data-driven value that corresponds to the mean score observed in the current study. It is not a standard value as no such cut-off was provided previously, and mean scores in different populations vary due to variations in demographic, cultural, and environmental attributes.

Regarding CFSS, a cut-off value of 44 was set, with scores above 44 indicating compromised cognition, while scores of 44 or below signify optimal cognition. The CFSS scale comprises 18 items, each rated on a five-point Likert scale, ranging from 1 ("never") to 5 ("always"). A higher score on the scale reflects greater impairment in cognitive functioning. The cut-off level of 44 in this study is a data-driven value that corresponds to the mean score (44.01±9.05) observed in the current study. No previous cut-off was provided for the classification of cognition.

A cutoff value of 3 was established for FFBQ, with a score of 3 or above indicating a fiber-rich diet or healthy diet, while a score below 3 reflects a fatty diet or unhealthy diet. The FFBQ is a 20-item instrument designed to assess dietary behaviors related to fat and fiber intake. The total index is calculated by dividing the total score with a number of responses. The score range is 1 (least healthy) to 5 (most healthy). A cutoff value of 3 was established, with scores of 3 or above indicating a fiber-rich diet or healthy behavior, while a score below 3 reflects a fat-rich diet or unhealthy behavior. This is according to the questionnaire guidelines and the mean score of this study, which is 2.76±0.53 [14].

A cutoff value of 16 was used for physical activity in this study. Subjects with a score equal or above 16 were classified as active, while those with a score below 16 were categorized as less active. Study data showed that a majority of students reported being physically active for more than four days a week at the workplace, with a daily duration between 10 and 15 minutes. Therefore, participants will accumulate 50-75 minutes/week (with a score of 8 to 10) from workplace activity. Consequently, the scoring cut-off of 16 was set to ensure the inclusion of this dominant trend as reflected by the mean level plus additional minutes from home or gym activity, yielding 100 to 120 minutes. According to WHO guidelines, a minimum of 150 minutes per week of moderate physical activity is required between 18 and 65 years of age [17-19]. A score of 16 was considered comparable for defining moderate activity in a young adult population in the current study.

For BMI, a cut-off level of 25 kg/m^2^ was set; and subjects with a BMI above 25 kg/m^2^ were obese, and those having a BMI equal or below it was non-obese. This cut-off was established according to the WHO guidelines for the Asian population, which stated that normal weight is between 18.5-22.9 kg/m^2^, overweight >23 kg/m^2^, and obese >25 kg/m^2^ [8].

In this study, the frequency of participants exhibiting optimal self-control was 46% (185/400), optimal cognition 51% (205/400), a preference for a fiber-rich diet 49% (197/400), and obesity 33% (130/400) (Table 2).

**Table 2:** Frequency of categorical variables. BMI: body mass index.

Categorical variables, Number=400	Frequency	Percentage
Gender		
Male	120	30
Female	280	70
Self-control		
Optimal	185	46
Sub-optimal	215	54
Cognitive ability		
Optimal	205	51
Sub-optimal	195	49
Dietary habits		
Fiber diet	197	49
Fat diet	203	51
Physical activity		
Moderate	228	57
Low	172	43
BMI-category		
Non-obese	270	67
Obese	130	33

The chi-square test was utilized to determine the association of categorical variables with each other (Table 3). There was a significant association of self-control with BMI, dietary behavior, and cognition (p<0.05). There was no significant association of self-control with physical activity (p>0.05). BMI showed a significant association with dietary behavior (p<0.05). However, there was no significant association of cognitive level with BMI, dietary behavior, and physical activity (p>0.05).

**Table 3:** Association of self-control and BMI with the study variables. Chi-square test is conducted for calculating the p-value and odds ratio (confidence interval). *A p-value less than 0.05 is of statistical significance. **Pearson chi-square value is represented alongside the p-value. Total number of participants is 400. N (%): number (percentage). BMI: body mass index.

Association of self-control with dietary habits					
Self-control	Fiber diet N (%)	Fat diet N (%)	Odds ratio (confidence interval)	Pearson chi-square value**	p-value
Optimal control N (%)	104 (26)	81 (20)	1.68 (1.13-2.50)	6.683	0.010*
Sub-optimal control N (%)	93 (23)	122 (31)			
Association of self-control with BMI categories					
	Non-obese	Obese	Odds ratio (confidence interval)	Pearson chi-square value**	p-value
Optimal control N (%)	140 (35)	45 (12)	2.05 (1.33-3.16)	10.487	0.001*
Sub-optimal control N (%)	130 (32)	85 (21)			
Association of self-control with cognition					
	Optimal cognition N (%)	Cognitive decline N (%)	Odds ratio (confidence interval)	Pearson chi-square value**	p-value
Optimal control N (%)	112 (28)	73 (18)	2.01 (1.35-3.00)	11.891	0.001*
Sub-optimal control N (%)	93 (23)	122 (31)			
Association of BMI with dietary habits					
BMI	Fiber diet N (%)	Fat diet N (%)	Odds ratio (confidence interval)	Pearson chi-square value**	p-value
Non-obese N (%)	143 (36)	127 (31)	1.59 (1.05-2.44)	4.582	0.03*
Obese N (%)	54 (14)	76 (19)			

Binary logistic regression was used to determine the association between BMI and different variables, including physical activity, self-control, dietary habits, and cognition. Potential confounding variables, such as age and gender, were controlled and included in the model. Self-control showed a significant association indicating that individuals with higher self-control are less likely to be obese (p=0.003). The dietary score showed a possible protective effect, but it was not statistically significant (p=0.07). Cognition and physical activity grade did not significantly impact the outcome variable BMI (p>0.05) (Table 4).

**Table 4:** Binary logistic regression analysis displaying the effect of different variables on the dependent variable BMI. Binary logistic regression analysis was conducted to identify significant variables influencing BMI. The dependent variable was BMI, while the co-variates included physical activity, self-control, dietary behavior, and cognition. Confounder variables, such as age and gender, were controlled in the model. *A p-value of less than 0.05 was considered statistically significant. Among these, self-control emerged as a significant variable, indicating that individuals with higher self-control are less likely to be obese (p<0.05). BMI: body mass index.

Co-variates	Beta coefficient	Exp (B) (odds ratio)	95% CI	p-value
Model-1				
Cognition	0.095	1.1	0.71-1.69	0.66
Physical activity	-0.107	0.89	0.58-1.38	0.62
Self-control	-0.693	0.50	0.32-0.78	0.002*
Dietary habits	-0.389	0.68	0.44-1.04	0.07
Model-2				
Self-control	-0.673	0.51	0.33-0.79	0.003*
Dietary habits	-0.394	0.67	0.44-1.03	0.07

## Discussion

This study evaluated the association of psychological factors such as self-control and cognition with lifestyle behaviors of dietary habits and physical activity. Self-control depicted a significant association with BMI and dietary preference and remained the key determinant of BMI after adjusting for confounder co-variates. This result is in alignment with a previous household survey in Australia, which found an association between limited self-control and body weight [20]. Another study from Korea reported self-control as a mediating factor in weight-related behavior among overweight and obese female students [21]. Seen in this context, it appears almost evident that most people require some level of self-control to maintain a healthy weight. Trait self-control refers to the ability to suppress immediate impulses effectively in order to prioritize and achieve long-term goals. The core dimensions of self-control as measured by BSCS include self-discipline, impulse management, and self-regulation of healthy behavior [9,10]. Self-control and self-regulation are often used interchangeably. However, self-control encompasses a situation of conflict, in which any temptation for a particular response is opposed by a competing desire. Dietary behavior means resisting urges to eat right away or stopping when no longer needed, despite craving to continue. This study also examined two key factors that may serve as potential links between self-control and BMI, including dietary restraint, which reflects the ability to regulate food intake to achieve a desired weight, and regular participation in physical activity [11]. There was no significant association of self-control with physical activity; however, a significant association was found between unhealthy dietary behavior and self-control in this study. Close intimacy between healthy dietary behavior and self-regulation has been reported by multiple previous studies [22,23]. Certainly, healthy dietary habits entail the capacity to resist the temptation to eat tasty food that contains refined sugar and is heavily caloric [2]. 

There was also a significant association of unhealthy dietary behavior with obesity in this study. This result is in concordance with the similar findings reported among students of two public sector medical colleges of Pakistan [24,25]. Another study on Pakistani adolescents and school-aged children unscored a significant association of body weight and obesity with nutritional behavior [26]. However, when the combined effect of all other factors influencing body weight was considered, this association of dietary preferences with BMI weakened and became non-significant. Only self-control emerged as the primary co-variate that significantly affected body mass index. In this study, the prevalence of obesity among students was 33% (130/400), which not only jeopardizes well-being by increasing the likelihood of stroke, diabetes, and cardiac derangements but also decreases economic prosperity by limiting efficiency, workplace engagement, and reducing earnings [20].

There was no association between cognition and BMI, dietary habits, or physical activity. This may be because CFSS, which predominantly evaluates memory, attention, and executive function, did not link with the aspects relevant to dietary and physical activity preference. Dietary choices and BMI relate more with behavioral and environmental factors. This finding is in concordance with the findings of the previous studies concluding that the association between dietary habits and executive functions is equivocal [27]. However, another study from India reported a positive correlation of cognition with BMI [28]. This aligns with the ongoing debate in the literature regarding the strength and consistency of this association, as various confounding factors often influence both cognitive performance and nutritional patterns. In the current study, cognitive functioning, as measured by CFSS, showed a significant association with self-control, suggesting a potential link between cognitive function and regulatory behavior. Participants with higher cognition exhibited better self-discipline, aligning with prior findings that executive functions, particularly attention and decision-making are critical to self-regulation [29].

There are certain limitations of this study. As it is a self-reported study, there may be potential bias in the data provided by the participants. In addition, the cross-sectional study design is restricted to establishing cause-and-effect relationships. The study has limited generalizability as it is conducted in a single institution.

## Conclusions

Self-control was identified as a significant variable influencing BMI, also exhibiting a notable association with dietary habits. Subjects with higher self-control are more likely to make healthy food choices, resist detrimental eating temptations and regulate their serving size, which ultimately leads to better weight management. In contrast, cognitive functioning and physical activity showed no association with BMI. These observations imply that cognition may influence perception, health literacy, or decision-making; however, it does not affect BMI status independently. Although physical activity is well documented to improve overall health, its direct impact on BMI may be influenced by other mediating factors such as metabolism and dietary intake. These results underscore the need for further research to unravel the intricate relationship between cognitive function, self-regulation, and health-related behaviors. 
