# Correction: Role of Self-Control and Cognitive Ability in Dietary Habits, Physical Activity, and Body Mass Index Among Medical Undergraduates

**DOI:** 10.7759/cureus.c221

**Published:** 2025-05-07

**Authors:** Uzma Zafar, Zaima Ali, Attiqa Khalid

**Affiliations:** 1 Physiology, Lahore Medical and Dental College, University of Health Sciences and University of Biological and Applied Sciences, Lahore, PAK

This article has been corrected to remove the research questionnaire, which includes the Cognitive Functioning Self-Assessment Scale (CFSS) and the Tangney Brief Self-Control Scale (BSCS). 

The CFSS was shared with the author for research purposes only, and the BSCS was similarly used under the understanding that it would not be publicly disseminated. These instruments were not intended for public sharing. 

The author sincerely regrets the oversight.

